# Supplementary material for: A combined computational and experimental investigation of the filtration function of splenic macrophages in sickle cell disease
Source: PLoS Comput Biol. 2023 Dec 13;19(12):e1011223. doi: 10.1371/journal.pcbi.1011223 (PMC10752522; doi:10.1371/journal.pcbi.1011223)
Supplement: S1 Text — (PDF) [file pcbi.1011223.s001.pdf]

A combined computational and experimental investigation of the filtration function of splenic macrophages in sickle cell disease

Guansheng Li, Yuhao Qiang, He Li, Xuejin Li, Pierre A. Buffet, Ming Dao and George Em Karniadakis

S1_Text. Hydrodynamics and particle-based blood cell models

In this work, we employ dissipative particle dynamics method (DPD) to model plasma, red blood cells (RBCs) and macrophages. The DPD method is a mesoscopic particle-based simulation technique, cwhere each DPD particle represents a lump of molecules and they interact with other particles through soft pairwise forces (1). DPD can provide the correct hydrodynamic behavior of fluids at the mesoscale, and it has been successfully applied to study complex fluids (2–4). The equation of motion for each particle i is governed by the sum of pair interactions $\mathbf{f}_i$ with the surrounding particles j and it is integrated using a velocity-Verlet algorithm. The time evolution of velocity ($\mathbf{v}_i$) and position ($\mathbf{r}_i$) of a particle i with mass m_i is determined by Newton's second law of motion:

$$\mathrm{d}\mathbf{r}_i = \mathbf{v}_i \,\mathrm{dt}\,; \quad \mathrm{d}\mathbf{v}_i = \mathbf{f}_i/m_i \,\mathrm{dt}. \tag{1}$$

In DPD method, the total force $\mathbf{f}_i$ exerted on particle i by particle j is composed of a conservative force ($\mathbf{F}_{ij}^C$), a dissipative force ($\mathbf{F}_{ij}^D$), and a random force ($\mathbf{F}_{ij}^R$) given by

$$\mathbf{F}_{ij}^C = a_{ij}(1 - \frac{r_{ij}}{r_c})\hat{\mathbf{r}}_{ij} \quad for \quad r_{ij} \le r_c; \quad 0 \quad for \quad r_{ij} > r_c, \tag{2}$$

$$\mathbf{F}_{ij}^D = \gamma\omega_d(r_{ij})(\hat{\mathbf{r}}_{ij} \cdot \hat{\mathbf{v}}_{ij})\hat{\mathbf{r}}_{ij}, \tag{3}$$

$$\mathbf{F}_{ij}^R = \sigma\omega_r(r_{ij})\frac{\zeta_{ij}}{\sqrt{dt}}\hat{\mathbf{r}}_{ij}, \tag{4}$$

where r_c is a cut-off radius, and a_{ij}, γ, σ are the conservative, dissipative, random coefficients, respectively, r_{ij} is the distance between two particles with the corresponding unit vector $\hat{\mathbf{r}}_{ij}$, $\hat{\mathbf{v}}_{ij}$ is the difference between the two velocities, ζ_{ij} is a Gaussian random number with zero mean and unit variance, and dt is the simulation timestep size. It is selected to be 0.001 in the current work. The parameters γ and σ and the weight functions coupled through the fluctuation-dissipation theorem and they are calculated by $\omega_d = \omega_r^2$ and $\sigma^2 = 2\gamma k_B T$, where k_B is the Boltzmann constant and T is the temperature of the system. The weight function $\omega_r(r_{ij}) = (1 - r_{ij}/r_c)^k$ with $k = 1$ in the standard DPD method, whereas other values of k have been used to increase the fluid viscosity (5**?**). The DPD parameters used in Eqs (2)-(4) for all types of DPD particles are given in Table S1.

In addition to blood plasma modeled by collections of free DPD particles, the membrane of suspending cells including RBCs and macrophages is constructed by a 2D triangulated network with N_v vertices (DPD particles). The vertices are connected by N_s elastic bonds to impose proper membrane mechanics. These DPD representations of RBCs and macrophages were extensively used and validated in the previous studies for both healthy and diseased cells (3**?** –6). For a single cell, the free energy (V_{cell}) is given by

$$V_{cell} = V_s + V_b + V_{a+v}. \tag{5}$$

Table S1: DPD parameters used in simulations. r_c is a cut-off radius, a_{ij} is the conservative coefficient, γ is the dissipative coefficient, and k is the weight function exponent. In all simulations, we set the particle mass $m = 1$, and the thermal energy $k_BT = 0.10$ in DPD units. Note that S: solvent (representing plasma), R: RBC, M: Macrophage.

type	r_c	a_{ij}	γ	k
S-S	1.0	5.0	30.0	0.25
S-R	1.5	5.0	30.0	0.25
S-M	1.5	5.0	30.0	0.25
R-R	1.0	10.0	30.0	0.25
R-M	1.0	10.0	30.0	0.25
M-M	1.0	10.0	30.0	0.25

The elastic energy V_s representing the elastic interactions of the cell membrane is defined by

$$V_s = \sum_{j\in 1...N_s} \left[\frac{k_{\mathrm{B}}Tl_m(3x_j^2 - 2x_j^3)}{4p(1-x_j)} + \frac{k_p}{l_j} \right], \tag{6}$$

where p is the persistence length, k_p is the spring constant, $k_{\mathrm{B}}T$ is the energy unit, l_j is the length of the spring j, l_m is the maximum spring extension, and $x_j = l_j/l_m$. p and k_p are computed by balancing the forces at equilibrium and from their relation to the macroscopic shear modulus, μ_s:

$$\mu_s = \frac{\sqrt{3}k_{\mathrm{B}}T}{4pl_mx_0}\left(\frac{x_0}{2(1-x_0)^3} - \frac{1}{4(1-x_0)^2} + \frac{1}{4}\right) + \frac{3\sqrt{3}k_p}{4l_0^3}, \tag{7}$$

where l_0 is the equilibrium spring length and $x_0 = l_0/l_m$. The bending resistance V_b of the cell membrane is modeled by

$$V_b = \sum_{j\in 1...N_s} k_b\left[1 - cos(\theta_j - \theta_0)\right], \tag{8}$$

where k_b is the bending constant, and it is related to the macroscopic bending rigidity k_c with the expression $k_b = 2k_c/\sqrt{3}$, θ_j is the instantaneous angle between two adjacent triangles sharing the common cedge j, and θ_0 is the spontaneous angle. In addition, the area and volume constraints V_{a+v} are imposed to mimic the area-preserving lipid bilayer and the incompressible interior fluid. The corresponding energy is given by

$$V_{a+v} = \sum_{j\in 1...N_t} \frac{k_d(A_j - A_0)^2}{2A_0} + \frac{k_a(A_{\mathrm{cell}} - A_0^{\mathrm{tot}})^2}{2A_0^{\mathrm{tot}}} + \frac{k_v(V_{\mathrm{cell}} - V_0^{\mathrm{tot}})^2}{2V_0^{\mathrm{tot}}}, \tag{9}$$

where N_t is the number of triangles in the membrane network, A_0 is the equilibrium value of a triangle area, and k_d, k_a and k_v are the local area, global area and volume constraint coefficients, respectively. The terms A_0^{tot} and V_0^{tot} are targeted cell area and volume.

We model RBCs with a biconcave shape using N_v=500 DPD particles. The shear modulus and bending rigidity of the RBC model are selected to be μ_0 = 4.73 μN/m and k_0 = 2.4×10^{-19} J, respectively. The cell surface area is selected to be A_0^{tot} = 132.9 μm^2, and cell volume V_0^{tot} = 92.5 μm^3, which give a surface to volume ratio of S/V = 1.44. All parameters used in our RBCs model are validated based on existing experimental data from single RBC mechanics to blood flow dynamics (3, 5**?** , 6). A passive macrophage

model is represented by a spherical cell with a radius of 6$\sim$ 11 μm, following our experimental observations. Macrophages are a subtype of white blood cells which are in general less deformable compared to RBCs, with an estimated shear modulus of 300$\sim$3000 μN/m (**?**) and bending stiffness of $1\sim2\times10^{-18}$ J (**?**). Guided by these studies, we model a macrophages with μ_s = 100 μ_0 and k_b = 8 k_0 in the current work.

The Morse potential model, was proposed by neglecting the physics behind the cell aggregation, in which a Morse potential function is used to fit the aggregation energy between two triangular elements on the cells. It has been widely accepted in many simulations of cells so far (**? ? ?**). In our work, we also adopt this simplified model, and the total aggregation energy is given by (**?**),

$$U_{agg} = \sum_{m=1,\cdots,N_t} \phi(r_{mm'})(n_m \cdot k_m)(n_{m'} \cdot k_{m'})A_m, \tag{10}$$

where the subscripts m and m' refer to the triangles m and m' of the two respective cells, n is the outward unit normal vector of the triangle, k is the unit vector in the direction parallel to the line joining the centers of two interacting cells, A_m is the area of the triangle m, and N_t is the number of triangles. Note that the terms $n_m \cdot k_m$ and $n_{m'} \cdot k_{m'}$ are introduced to describe the interaction between two curved surfaces instead of flat planes (**?**). $\phi(r_{mm'})$ is the Morse potential between two facing unit flat planes, given by (**?**),

$$\phi(r_{mm'}) = E_I \left[e^{2\beta(r_0 - r_{mm'})} - 2e^{\beta(r_0 - r_{mm'})} \right], \tag{11}$$

where E_I is a surface energy, β is a scaling factor, r_0 is a zero force separation, $r_{mm'}$ is the local distance between two facing unit flat planes. This aggregation model describes a strong repulsion at a near intercellular distance, but a weak attraction at a far intercellular distance.

References

1. R. D. Groot and P. B. Warren. Dissipative particle dynamics: Bridging the gap between atomistic and mesoscopic simulation. *The Journal of Chemical Physics*, 107:4423–4435, 1997.

2. T. Ye, T. N. Phan, and C. T. Lim. Particle-based simulations of red blood cells –a review. *Journal of Biomechanics*, 49:2255–2266, 2016.

3. H. Lei and G. E. Karniadakis. Probing vasoocclusion phenomena in sickle cell anemia via mesoscopic simulations. *Proc. Natl. Acad. Sci.*, 110:11326–11330, 2013.

4. H. Chang, A. Yazdani, X. Li, K. Douglas, C. S. Mantzoros, and G. E. Karniadakis. Quantifying platelet margination in diabetic blood flow. *Biophysical Journal*, 115:1371–1382, 2018.

5. D. A. Fedosov, B. Caswell, and G. E. Karniadakis. A multiscale red blood cell model with accurate mechanics, rheology, and dynamics. *Biophysical Journal*, 98:2215–2225, 2010.

6. I. V. Pivkin and G. E. Karniadakis. Accurate coarse-grained modeling of red blood cells. *Physical Review Letters*, 101:118105, 2008.
