# Supplementary material for: A combined computational and experimental investigation of the filtration function of splenic macrophages in sickle cell disease
Source: PLoS Comput Biol. 2023 Dec 13;19(12):e1011223. doi: 10.1371/journal.pcbi.1011223 (PMC10752522; doi:10.1371/journal.pcbi.1011223)
Supplement: S2 Text — (PDF) [file pcbi.1011223.s002.pdf]

A combined computational and experimental investigation of the filtration function of splenic macrophages in sickle cell disease

Guansheng Li, Yuhao Qiang, He Li, Xuejin Li, Pierre A. Buffet, Ming Dao and George Em Karniadakis

S2_Text. Details of experiments

Sickle blood samples were drawn from homozygous sickle cell disease patients at the Massachusetts General Hospital under an Excess Human Material Protocol approved by the Partners Healthcare Institutional Review Board (IRB) with a waiver of consent. The microfluidic experiments were performed under an approved exempt protocol at the Massachusetts Institute of Technology (IRB protocol E-1523). Three HbSS blood samples were collected for the experiments. The complete blood count (CBC) and hemoglobin electrophoresis data for all HbSS blood samples used in our experiments are summarized in Tab. S1. Complete blood count (CBC) data: the white blood count (WBC), hematocrit (HCT), mean corpuscular volume (MCV), and mean corpuscular hemoglobin concentration (MCHC). Hemoglobin electophoresis data: the fraction (%) of hemoglobin S (HbS), hemoglobin F (HbF), hemoglobin A (HbA), and hemoglobin A2 (HbA2).

Table S1: Clinical Measurements of sickle cell blood samples.

Patient	Genotype	WBC	HCT	MCV, fl	MCHC, g/dl	HU	HbS	HbF	HbA	HbA2
Patient1	HbSS	9.49	36.4	71.9	36.5	N	50.9%	1.3%	0.0%	4.4%
Patient2	HbSS	10.25	19.2	102.1	33.3	Y	86.0%	9.6%	0.0%	4.4%
Patient3	HbSS	10.76	23.3	96.7	32.2	Y	49.3%	1.2%	3.3%	3.5%
