# Supplementary material for: A combined computational and experimental investigation of the filtration function of splenic macrophages in sickle cell disease
Source: PLoS Comput Biol. 2023 Dec 13;19(12):e1011223. doi: 10.1371/journal.pcbi.1011223 (PMC10752522; doi:10.1371/journal.pcbi.1011223)
Supplement: S3 Text — (PDF) [file pcbi.1011223.s003.pdf]

A combined computational and experimental investigation of the filtration function of splenic macrophages in sickle cell disease

Guansheng Li, Yuhao Qiang, He Li, Xuejin Li, Pierre A. Buffet, Ming Dao and George Em Karniadakis

S3_Text. Calibrate the adhesion strength K_s based on microfluidic experiments

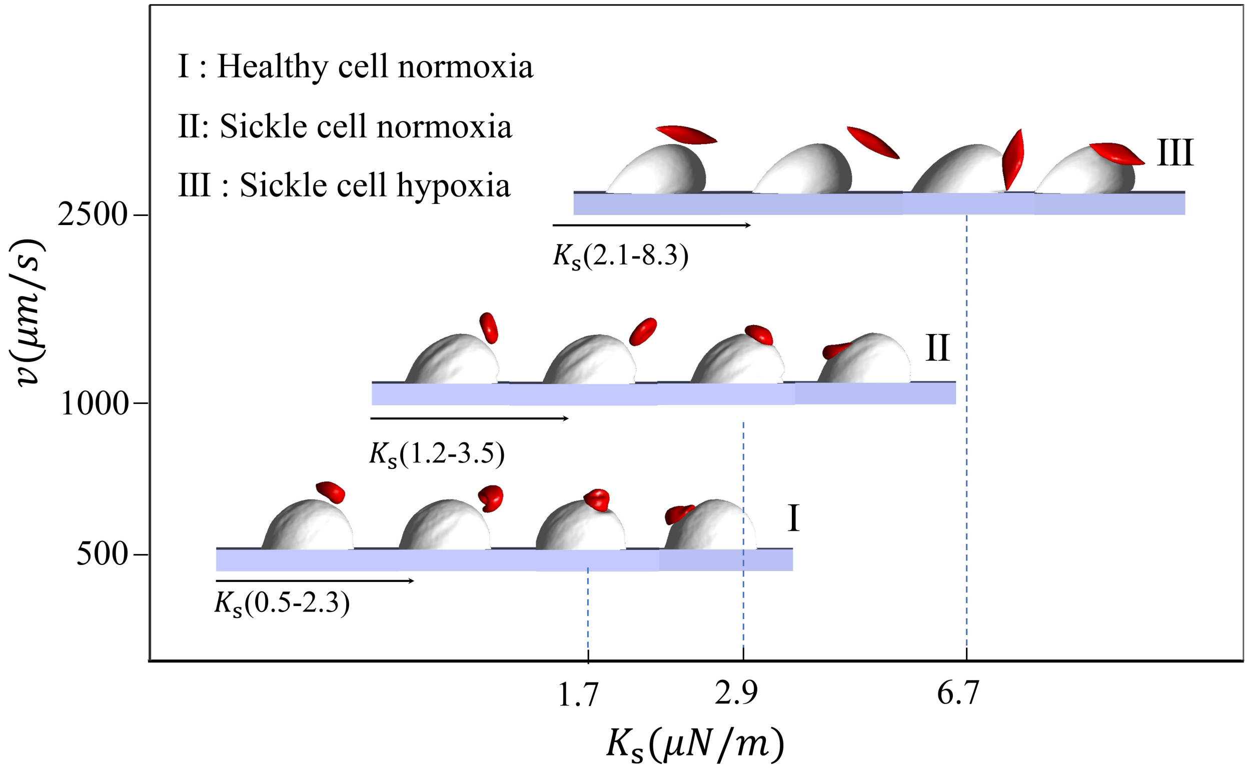

Figure S1: Calibrate the parameter K_s of stochastic adhesion model through experimentation. The detachment dynamics are performed to obtain the critical K_s that corresponds to the various critical velocities: healthy cell under normoxia ($500 mum/s$), sickle cell under normoxia ($1000 mum/s$), and sickle cell under hypoxia ($2500 mum/s$).

We investigate the adhesion dynamics of normal and sickle RBCs in Coette flow under both normoxia and hypoxia, inferring the cell adhesion strength K_s by increasing the flow velocity until the onset of cell detachment. Specifically, we determine the K_s value by fitting the experimental flow velocity values required for cell detachment. A greater K_s will contribute to a stronger cell adhesion strength, thus resulting in a firm adhesion and a high flow velocity is required for cell detachment, while the cell will be easily detached from the adherent surface with a smaller K_s. Fig. S1 depicts the process of determining K_s in the adhesion model. We perform the simulation to mimic the detachment dynamics of RBCs from macrophages and try to identify

the critical K_s that leads to the onset of detachment after the RBCs make contact with the macrophages. All the detachment dynamics are investigated at the critical detachment velocities measured from the microfluidic experiments. Three kinds of conditions are investigated in Fig. S1, including normal cells under normoxia(I), sickle cells under normoxia(II) and sickle cells under hypoxia(III). Our simulation results show that when we increase the K_s from $0.5\mu N/m$ to $1.7\mu N/m$ at the critical velocity $500\mu m/s$ for normal cell under normoxia, the cell will detach from the macrophages. When K_s greater than $1.7\mu N/m$, the RBCs will form a flipping or firm adhesion. Thus, we select $K_s = 1.7\mu N/m$ for normal cell under normoxia. In a similar manner, we obtain $K_s = 2.9\mu N/m$ and $K_s = 6.7\mu N/m$ for sickle cell under normoxia and hypoxia states, respectively.
