# Supplementary material for: A combined computational and experimental investigation of the filtration function of splenic macrophages in sickle cell disease
Source: PLoS Comput Biol. 2023 Dec 13;19(12):e1011223. doi: 10.1371/journal.pcbi.1011223 (PMC10752522; doi:10.1371/journal.pcbi.1011223)
Supplement: S4 Text — (PDF) [file pcbi.1011223.s004.pdf]

A combined computational and experimental investigation of the filtration function of splenic macrophages in sickle cell disease

Guansheng Li, Yuhao Qiang, He Li, Xuejin Li, Pierre A. Buffet, Ming Dao and George Em Karniadakis

S4_Text. Calibrate the bond formation and dissociation k_{on}^0, k_{off}^0 and the sensitivity analysis of the parameters

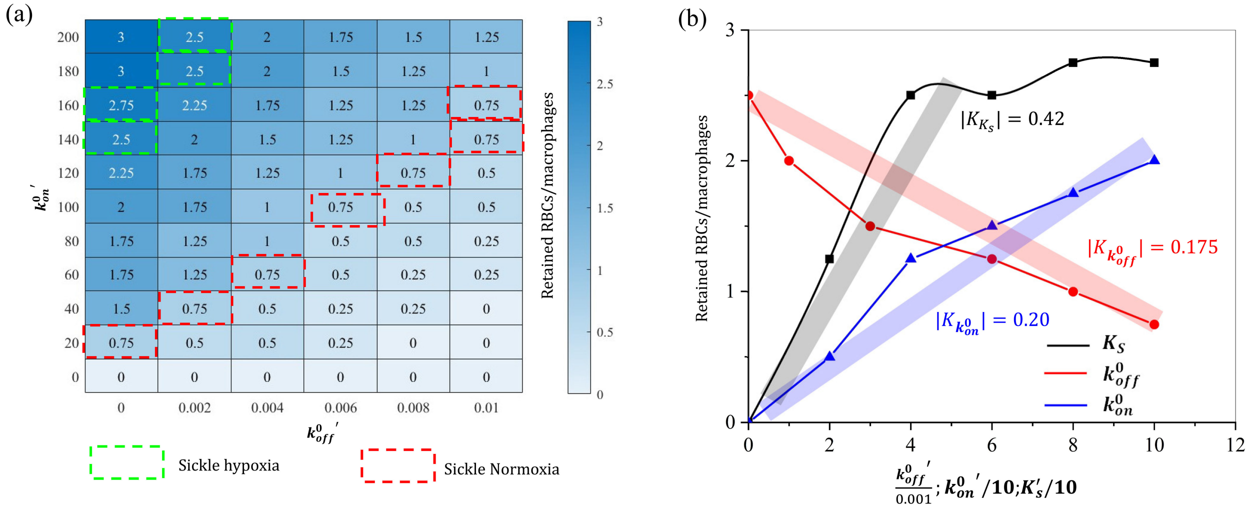

Figure S1: The calibration process of the k_{on}^0 and k_{off}^0 in the stochastic model. (a) The average number of retained RBCs per macrophage varies with k_{on}^0 and k_{off}^0. (b) The parameter sensitivity analysis for the sickle cell under hypoxia encompasses the parameters k_{on}^0, k_{off}^0, and K_S.

Figure S1(a) presents the process of calibrating the parameters governing bond formation and dissociation, namely, k_{on}^0 and k_{off}^0. The variation in colors within the figure signifies the dependence of the number of RBCs retained on individual macrophages on the values of k_{on}^0 and k_{off}^0. To ascertain the appropriate parameter values, a comparative analysis was conducted, aligning the number of RBCs retained on macrophages between experimental data and simulations. The experimental results revealed that, under hypoxic and normoxic conditions, the average number of RBCs retained per macrophage stood at approximately 2.7 and 0.75, respectively. Consequently, adjustments were made to the model's parameters to match the simulated number of RBCs retained per macrophage. Drawing inspiration from our previous research detailed in (1), it was observed that a higher bond formation rate k_{on}^0, and a lower bond break rate k_{off}^0, fostered the formation of bonds between RBCs and macrophages. During the calibration process, a fixed value of $K_s = 3.36\mu N/m$(corresponding simulation parameter $K_s^{'} = 70$) was selected, while the physical parameters k_{on}^0 were adjusted within the range of $0 \sim 10.84 * 10^4 s^{-1}$ and k_{off}^0 within the range of $0 \sim 5.42 s^{-1}$, respectively, in accordance with literature-reported values. In contrast, the simulation values, denoted as ${k_{on}^0}^{0}$ and ${k_{off}^0}^{0}$, spanned the ranges of $0 \sim 200$ and $0 \sim 0.01$, respectively. Within Figure S1(a), the green frame

delineates the suitable parameters for the hypoxic condition, while the red frame represents the parameters suitable for the normoxic condition. Consequently, we choose unique parameters from the two sets corresponding to various conditions, including $k^0_{on} = 5.42 * 10^4 s^{-1}$ and $k^0_{off} = 2.71 s^{-1}$ for normoxia condition, $k^0_{on} = 8.14 * 10^4 s^{-1}$ and $k^0_{off} = 0.54 s^{-1}$ for hypoxia condition.

Figure S1(b) illustrates the sensitivity analysis of the parameters K_s, K^0_{on}, and k^0_{off} under hypoxic condition. This analysis involved keeping two of the three parameters constant while increasing one to observe its impact on the number of RBCs retained on macrophages. To facilitate the comparison of the three parameters, a standardization process is utilized to scale each parameter within the range of 0 to 10. The findings indicated that the number of RBCs retained on macrophages initially increased as $K^{'}_s$ decreased below 60, eventually reaching a plateau. Similarly, an increase in k^0_{on} positively correlated with RBC adhesion, while an increase in k^0_{off} exhibited the opposite effect. To quantitatively compare the sensitivity of these three parameters, we computed the absolute values of the curve slopes, revealing that the sensitivity ranking among these parameters was $K_s > k^0_{on} > k^0_{off}$.

References

1. Y. Deng, D. P. Papageorgiou, H. Chang, S. Z. Abidi, X. Li, M. Dao, and G. E. Karniadakis. Quantifying shear-induced deformation and detachment of individual adherent sickle red blood cells. *Biophys. J.*, 116:360–371, 2019.
