# Supplementary material for: A combined computational and experimental investigation of the filtration function of splenic macrophages in sickle cell disease
Source: PLoS Comput Biol. 2023 Dec 13;19(12):e1011223. doi: 10.1371/journal.pcbi.1011223 (PMC10752522; doi:10.1371/journal.pcbi.1011223)
Supplement: S5 Text — (PDF) [file pcbi.1011223.s005.pdf]

A combined computational and experimental investigation of the filtration function of splenic macrophages in sickle cell disease

Guansheng Li, Yuhao Qiang, He Li, Xuejin Li, Pierre A. Buffet, Ming Dao and George Em Karniadakis

S5_Text. Accelerated time sale

To align with the experiment's timeframe, we employ an accelerated time scale in simulation. Specifically, within the framework of the stochastic adhesion model, the formation rate k_{on}^0 is related to time, and $k_{on}^0 \in (0.1, 100) * 10^4 s^{-1}$, so we choose $k_{on}^0 = 5.42 * 10^3 s^{-1} \in (0.1, 100) * 10^4 s^{-1}$, and the DPD value of ${k_{on}^0}' = 100$, so we can get an accelerated DPD time unit $\tau_a = 1.8437 * 10^{-2} s$, which is 10 times of the primary DPD time unit $\tau = 1.8437 * 10^{-3} s$. As a result, the adhesion time scale is comparable to the experimental findings. Additionally, we quantified the progression of sickle cell filtration by macrophages over time in sections 4.2 and 4.3 of the manuscript.
